# Cross-Domain Generalization of a CNN Trained on Oral and Oropharyngeal Squamous Cell Carcinoma Histopathology Using External Validation on Metastatic Lymph Node Tissue

**DOI:** 10.3390/cancers18172752

**Published:** 2026-08-25

**Authors:** Lorena Adriana Paun, Iulian Alexandru Taciuc, Mihai Dumitru, Daniela Vrinceanu, Andreea Marinescu, Alexandru-Darius Dragomir-Serboiu, Alina Oancea, Monica-Mihaela Cirstoiu, Adrian Costache

**Affiliations:** 1 ENT Department, Carol Davila University of Medicine and Pharmacy, 020021 Bucharest, Romania; paunlorena98@yahoo.com (L.A.P.); vrinceanudana@yahoo.com (D.V.); alexandru-darius.dragomir2024@stud.umfcd.ro (A.-D.D.-S.); 2 Pathology Department, Carol Davila University of Medicine and Pharmacy, 020021 Bucharest, Romania; adriancostacheeco@yahoo.com; 3 Radiology and Imaging Department, Carol Davila University of Medicine and Pharmacy, 020021 Bucharest, Romania; andreea_marinescu2003@yahoo.com; 4 ENT Department, Elias Clinical Hospital, 011461 Bucharest, Romania; dr.alina.oancea@gmail.com; 5 Gynecology Department, Carol Davila University of Medicine and Pharmacy, 020021 Bucharest, Romania; monica.cirstoiu@umfcd.ro

**Keywords:** oral squamous cell carcinoma, oropharyngeal squamous cell carcinoma, histopathology, convolutional neural network, transfer learning, cross-domain generalization, lymph node metastasis

## Abstract

**Background:** Convolutional neural networks (CNNs) perform well in histopathological classification of oral squamous cell carcinoma (OSCC), but their robustness across distinct tissue domains remains insufficiently studied. This study assessed whether a CNN trained only on primary oral and oropharyngeal squamous cell carcinoma images remained transferable to an independent metastatic lymph node histopathology dataset. **Methods:** Three public datasets containing 14,760 OSCC/OPSCC and 8530 normal oral mucosa images were combined for model development. An ImageNet-pretrained EfficientNetB0 backbone was used as a fixed feature extractor with a task-specific binary classification head. Performance was first assessed on an independent internal testing subset and subsequently evaluated on 20,000 H&E-stained normal and metastatic lymph node patches from a separate public dataset. **Results:** The model achieved an internal testing accuracy of 91.48%, with 92.95% sensitivity, 88.92% specificity, 93.56% precision, a 93.25% F1-score, and a Youden’s J index of 0.819. External validation resulted in a substantial decrease in overall classification performance, with an accuracy of 56.91%, specificity of 33.32%, precision of 48.71%, F1-score of 63.37%, balanced accuracy of 61.99%, MCC of 0.279, and a Youden’s J index of 0.240. Nevertheless, sensitivity for metastatic tissue remained high at 90.66%, indicating a markedly asymmetric external error profile characterized predominantly by false-positive classifications. **Conclusions:** The marked performance decrease during external validation demonstrates the limitations of direct cross-domain transfer between substantially different histopathological environments. However, the preserved sensitivity suggests that some discriminative information remained transferable beyond the development domain. These findings support partial rather than universal cross-domain generalization and emphasize the importance of independent out-of-distribution evaluation when assessing deep learning robustness in computational pathology.

## Simple Summary

Artificial intelligence is increasingly used to help analyze microscope images of cancer, but it is important to know whether these systems still work when tested on tissue that differs from the images used for training. In this study, we trained a computer model to distinguish oral and oropharyngeal squamous cell carcinoma from normal tissue using publicly available microscope images. We then tested whether the model could recognize cancer in a separate set of lymph node tissue images containing metastatic disease. The model performed well on images similar to those used during training. When tested on lymph node tissue, its overall accuracy decreased, showing that differences in tissue type and image source strongly affect performance. However, the model still detected most metastatic tissue correctly. These findings suggest that artificial intelligence can learn some cancer-related microscopic features that transfer across tissue types, but careful external testing is essential before clinical use.

## 1. Introduction

Oral squamous cell carcinoma (OSCC) and oropharyngeal squamous cell carcinoma (OPSCC) are among the most common malignancies of the head and neck region, with squamous cell carcinoma accounting for more than 90% of all malignant epithelial tumors arising at these anatomical sites [1]. Collectively, head and neck squamous cell carcinomas (HNSCC) represent a major global health burden, with oropharyngeal cancer ranking among the most prevalent cancers worldwide [2].

The incidence of both OSCC and OPSCC has increased over recent decades, particularly among younger individuals under the age of 45 years [3]. Although tobacco and alcohol consumption remain the principal etiological factors, infection with high-risk human papillomavirus (HPV), particularly HPV-16, has emerged as an important risk factor, especially for OPSCC [4]. HPV infects the basal keratinocytes of the stratified squamous epithelium through microscopic abrasions of the mucosal surface. Persistent infection may lead to viral genome integration and expression of the E6 and E7 oncoproteins, promoting malignant transformation through inactivation of the p53 and retinoblastoma (Rb) tumor suppressor pathways [5].

Although OSCC and OPSCC are frequently discussed together because of their common squamous cell origin, they constitute biologically distinct entities. HPV-driven carcinogenesis is a defining feature of a large subset of OPSCCs, whereas conventional OSCC is generally considered HPV-independent, with only a small proportion of tumors demonstrating evidence of transcriptionally active HPV infection [6].

Histopathological examination remains the gold standard for the diagnosis of OSCC and OPSCC. Conventional OSCC is the predominant histological subtype; however, several uncommon variants have also been described, including acantholytic, basaloid, sarcomatoid (spindle cell), papillary, and verrucous squamous cell carcinoma [7]. These variants exhibit distinct biological behavior and prognostic significance. While basaloid, sarcomatoid, and acantholytic variants are generally associated with a more aggressive clinical course and poorer prognosis, verrucous squamous cell carcinoma typically demonstrates a more favorable outcome because of its locally invasive but rarely metastatic nature [7,8].

The characteristic histopathological features of conventional OSCC include invasive nests, islands, cords, or sheets of atypical squamous epithelial cells infiltrating the underlying stroma, varying degrees of keratinization with keratin pearl formation, intercellular bridges, nuclear pleomorphism, hyperchromasia, increased mitotic activity, dyskeratosis, stromal desmoplasia, and variable inflammatory cell infiltration [9]. Tumor differentiation ranges from well- to poorly differentiated lesions, with decreasing keratinization and increasing cellular atypia accompanying higher histological grades. Recent studies have also demonstrated that HPV-negative tumors tend to exhibit increased tumor-associated neutrophil (TAN) infiltration compared with HPV-positive tumors, reflecting differences in the tumor immune microenvironment [4]. Furthermore, muscle invasion has been reported in approximately 70% of OSCC cases, while perineural invasion is observed in nearly 38% of patients, both representing adverse prognostic factors [8]. Several studies have shown that specific patterns of invasion correlate with disease progression, lymph node metastasis, and patient survival [10]. Primary tumors frequently invade as cohesive nests, islands, or cords of malignant squamous cells, often with focal keratin pearl formation, and these architectural patterns are commonly preserved within metastatic lymph nodes [11]. Nevertheless, histological differentiation may change during metastatic progression, as well-differentiated primary tumors can exhibit moderate or poor differentiation within lymph node metastases, increasing diagnostic complexity [12].

Recent advances in artificial intelligence (AI), particularly convolutional neural networks (CNNs), have demonstrated considerable potential for assisting histopathological diagnosis. However, the extremely large size of whole-slide images (WSIs), often reaching several gigapixels, poses substantial computational and storage challenges. Consequently, early studies utilizing a limited number of WSIs reported only moderate segmentation performance, with Intersection over Union (IoU) values ranging from 0.69 to 0.78 [13]. To overcome these limitations, a patch-based approach has become the predominant strategy in computational pathology. By extracting smaller image patches from WSIs, substantially larger training datasets can be generated while reducing computational requirements and enabling efficient binary or multiclass classification tasks [14].

Despite the growing number of AI studies in computational pathology, most CNN-based models are trained and evaluated on datasets originating from the same institution, scanner, staining protocol, or anatomical site. Consequently, their ability to generalize across different laboratories, acquisition protocols, and biologically distinct tissue environments remains insufficiently explored [15]. In particular, little is known about whether morphological features learned by AI from primary squamous cell carcinomas can be successfully transferred to metastatic deposits within lymph nodes originating from heterogeneous primary malignancies.

Therefore, the present study investigates the generalization capability of an EfficientNet-based convolutional neural network trained on three independent datasets of OSCC and OPSCC histopathological images. The trained model was subsequently evaluated on a fourth, completely independent dataset consisting of lymph node metastases. Because all datasets were acquired at different institutions using different scanners, staining protocols, and digitization workflows, this experimental design represents a stringent out-of-distribution (OOD) generalization setting.

We hypothesize that a convolutional neural network trained exclusively on primary oral and oropharyngeal squamous cell carcinoma histopathology can partially generalize to a distinct histopathological domain by learning transferable morphological representations rather than relying solely on dataset- or scanner-specific image characteristics. Therefore, beyond evaluating conventional classification performance, the primary objective of this study was to investigate the extent to which histopathological features learned from primary squamous cell carcinomas can be transferred to an independent out-of-distribution dataset of metastatic lymph node tissue. Furthermore, the study seeks to provide insight into the morphological representations captured by deep learning models and their potential robustness across different histopathological domains. Unlike previous studies that primarily focus on maximizing classification accuracy within a single histopathological domain, the present work aims to investigate whether the learned representations remain transferable when the model is challenged with a fundamentally different tissue type under true out-of-distribution conditions.

## 2. Materials and Methods

### 2.1. Database

Four publicly available histopathological datasets obtained from the Kaggle platform were included in this study. The first three datasets were used for model development (training, validation, and internal testing), whereas the fourth independent dataset was reserved exclusively for external validation to assess the model’s cross-dataset generalizability. The datasets comprise hematoxylin and eosin (H&E)-stained microscopic images obtained from oral and oropharyngeal lesions. Although the acquisition protocols varied among datasets, all images represented histopathological tissue samples suitable for the identification of squamous cell carcinoma.

First dataset [16,17]—This dataset consists of microscopic images of normal oral epithelium and oral squamous cell carcinoma (OSCC). The original dataset contained 1224 images, which were subsequently augmented to generate 2634 OSCC images and 2494 normal images. Image acquisition was performed using a Leica DM750 microscope (Leica Microsystems, Wetzlar, Germany) equipped with an ICC50 HD digital camera at 100× and 400× magnification. Images were acquired at a resolution of 2084 × 1536 pixels and stored in JPEG format.

Second dataset [18]—This multicancer histopathological dataset includes images from eight different malignancies. Only the OSCC, OPSCC, and corresponding normal tissue images were extracted for the present study, resulting in a balanced dataset of 2501 malignant and 2501 normal images (5002 images in total). Unlike whole-slide imaging (WSI) datasets, this collection consists of fixed-size (512 × 512 pixel) image patches without accompanying metadata regarding the acquisition system, magnification, patient cohort, or staining protocol. Visual inspection suggests that the images were acquired using a conventional microscope-mounted digital camera rather than a whole-slide scanner, as indicated by the limited field of view, variable color balance, and non-uniform illumination. In addition, characteristic banding artifacts were consistently observed along the image borders, likely originating from image acquisition or compression rather than representing true histopathological features. Because these artifacts occurred with comparable frequency in both malignant and normal classes, they were considered unlikely to introduce systematic class-specific bias. Nevertheless, the absence of acquisition metadata and the presence of imaging artifacts represent important limitations of this dataset. To minimize the influence of dataset-specific characteristics and improve model robustness, this dataset was combined with two additional independent histopathological datasets during model development.

Third dataset [19]—This dataset comprised 9129 malignant images (OSCC and OPSCC) and 1035 normal images acquired as high-resolution microscopic photographs (2592 × 1944 pixels, JPEG format) at 400× magnification. Unlike WSI-derived image patches, these images correspond to complete microscopic fields acquired directly through conventional microscopy. The dataset was organized into multiple histopathological categories, including normal mucosa, well-differentiated squamous cell carcinoma (WDSCC), moderately differentiated squamous cell carcinoma (MDSCC), poorly differentiated squamous cell carcinoma (PDSCC), and oral submucous fibrosis (OSMF). However, no additional information regarding the microscope model, acquisition protocol, imaging system, or patient cohort was provided by the dataset authors.

Fourth dataset [20]—An independent lymph node metastasis dataset containing 277,793 image patches from normal and metastatic lymph nodes was used exclusively for external validation. The external validation dataset consisted of 96 × 96-pixel image patches extracted from H&E-stained whole-slide images of sentinel lymph node tissue and labeled for the presence or absence of metastatic tumor deposits. The dataset represents the publicly available Histopathologic Cancer Detection (PatchCamelyon) dataset derived from the CAMELYON16 challenge. The original slides were digitized at two medical centers using a 40× objective (approximately 0.243 μm/pixel) and different scanner systems, including the 3DHistech Pannoramic Flash II 250 (3DHISTECH Kft., Öv street 3, H-1141, Budapest, Hungary) and Hamamatsu NanoZoomer-XR C12000-01 (Hamamatsu Photonics K.K., Sunayama-cho 325-6, Chuo-ku, Hamamatsu, Shizuoka, 430-8587, Japan) [21]. For the present study, 20,000 randomly selected labeled images from the training partition were used for testing after random shuffling. This dataset was intentionally excluded from model training to provide an independent assessment of model generalizability.

Overall, the first three datasets contributed 14,760 malignant (OSCC/OPSCC) and 8530 normal oral and oropharyngeal mucosal images for model development (Table 1). The malignant cohort included well-, moderately-, and poorly differentiated squamous cell carcinomas. Since the objective of the study was binary discrimination between malignant and non-malignant tissue rather than histological grading, all squamous cell carcinoma subtypes were merged into a single positive class during model training.

### 2.2. Study Workflow

**Figure 1:**
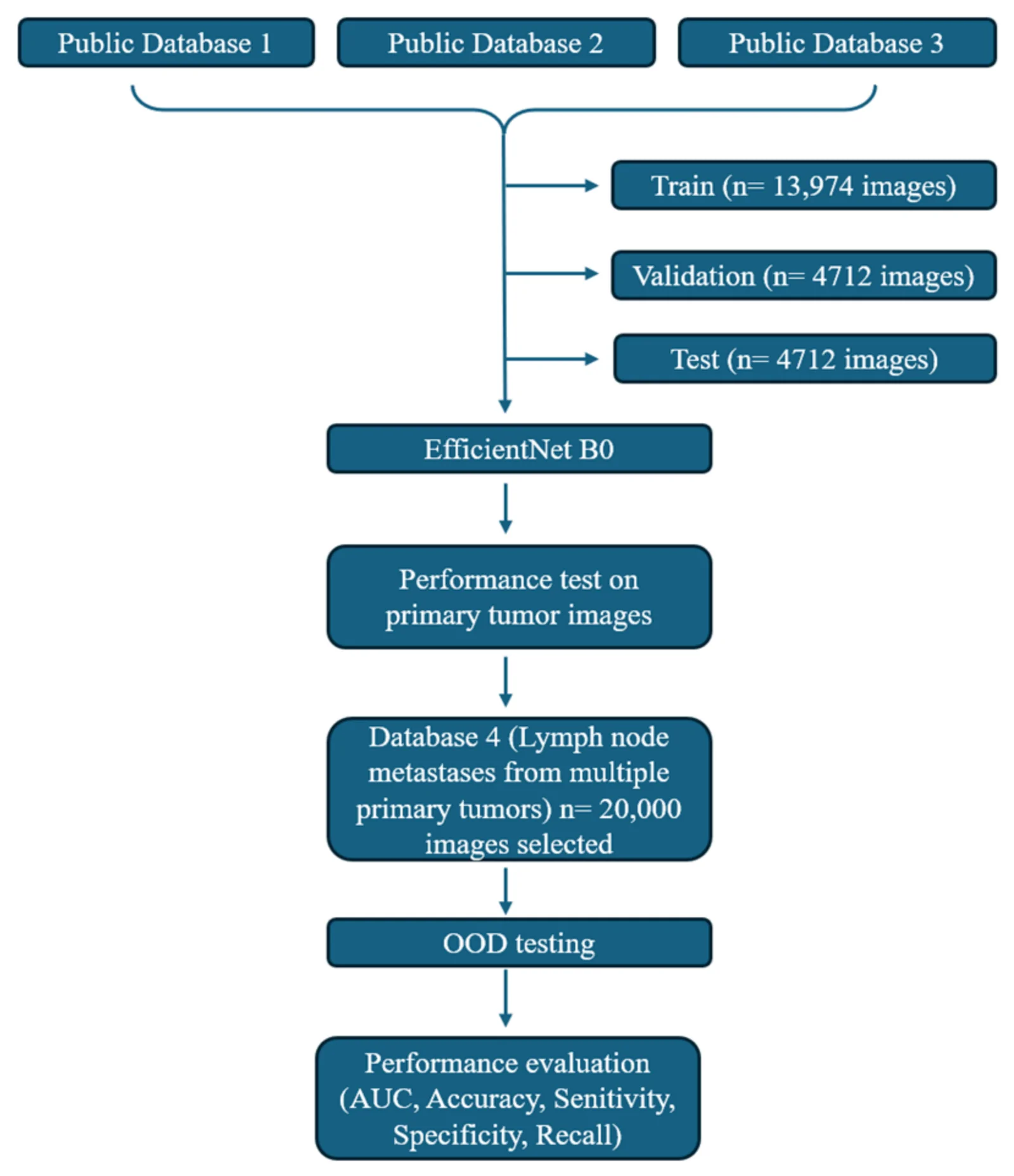
Overview of the study workflow.

The overall study workflow is presented in Figure 1. The first three publicly available datasets were combined and used for model development, including training, validation, and internal testing. To evaluate model robustness and generalizability, the final model was subsequently assessed on an independent external dataset that was not involved in any stage of model training, hyperparameter optimization, or model selection. This dataset originated from different institutions and image acquisition workflows and was therefore considered an out-of-distribution (OOD) test set, providing a realistic assessment of the model’s ability to generalize to previously unseen histopathological data.

### 2.3. Labeling and Data Checking

All included datasets provided binary image annotations (0 = normal, 1 = malignant). A visual quality assessment was performed by randomly inspecting representative images from each dataset to verify label consistency and identify potential annotation errors or corrupted images. No discrepancies or obvious labeling inconsistencies were identified; therefore, no images were excluded from further analysis. This assessment was not intended as a tissue-content filtering procedure, and images were not excluded solely because of limited tissue representation.

### 2.4. Model Implementation and Image Preprocessing

The proposed model was integrated and adapted using the Python (version 3.12) programming language, using Jupyter Notebook on the Kaggle platform. The training process was conducted within the same platform, which offers access to NVIDIA Tesla P100 graphics cards and Intel Xenon 2.3 GHz processors and 13 GB RAM.

To evaluate the study hypothesis, an EfficientNetB0 architecture [22] pretrained on ImageNet (https://www.image-net.org/about.php; accessed on 4 August 2026) was selected as the backbone network. The original classification head was removed, allowing the network to function exclusively as a feature extractor. A custom binary classification head was subsequently added. First, a GlobalAveragePooling2D layer converted the extracted feature maps into a compact feature vector. This was followed by a fully connected layer containing 128 neurons with a Rectified Linear Unit (ReLU) activation function to learn discriminative relationships among the extracted features. Two Dropout layers were incorporated before and after the dense layer to reduce the risk of overfitting. Finally, a single-neuron output layer with a sigmoid activation function generated the probability of malignant tissue.

EfficientNetB0 was initialized with ImageNet-pretrained weights and used as a fixed feature extractor. Although ImageNet contains natural rather than histopathological images, convolutional pretraining provides generic visual representations related to edges, gradients, textures, local shapes, and spatial patterns that can be reused for downstream image classification tasks. In the present study, these pretrained representations were retained without fine-tuning, while a task-specific classification head was trained on the histopathological datasets.

Prior to training, all images were resized to 96 × 96 pixels using bilinear interpolation to match the input dimensions of the external validation dataset. No additional handcrafted feature extraction was performed, allowing the convolutional neural network to automatically learn relevant histomorphological representations directly from the image data. All images were resized to 96 × 96 pixels to ensure compatibility with the external validation dataset while maintaining a uniform input size across all datasets. No additional tissue-content filtering was applied to the external validation subset before inference, allowing the model to be evaluated on the image distribution provided by the source dataset.

**Figure 2:**
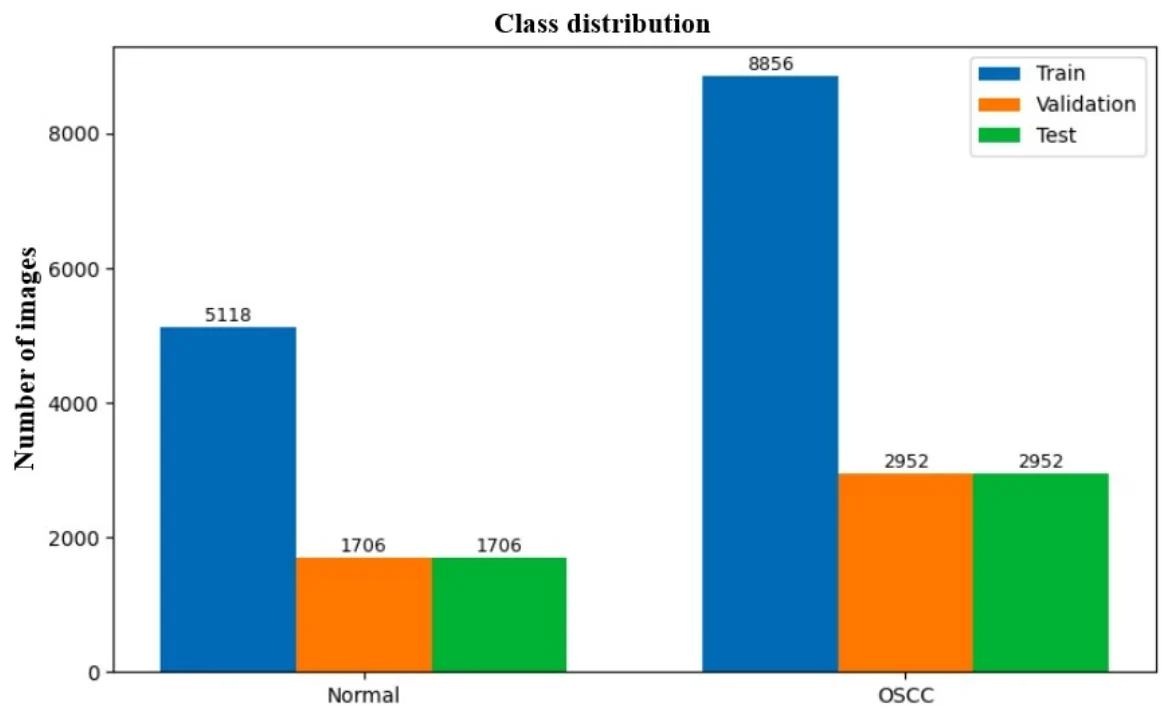
Distribution of images across the training, validation, and internal testing subsets. 60% of images were used for training, 20% for validation and 20% for internal testing. These images do not include the 20,000 patches from the lymph nodes database. The code used is available as Supplementary Material File S1.

The first three datasets were merged and randomly divided into training, validation, and internal testing subsets (Figure 2). This initial evaluation was intentionally performed using images acquired under heterogeneous but partially overlapping acquisition conditions to establish baseline classification performance before assessing model generalization on the independent out-of-distribution dataset.

Training was performed using the Adamax optimizer with a binary cross-entropy loss function, which is appropriate for binary classification with a sigmoid output layer. The ImageNet-pretrained EfficientNetB0 backbone was kept frozen throughout training and functioned as a fixed feature extractor. Therefore, optimization during the 10 training epochs was restricted to the newly added task-specific classification layers. Two Dropout layers (rates of 0.30 and 0.20) were used for regularization and were active exclusively during training.

## 3. Results

### 3.1. Performance of the Proposed Model on the Development Datasets

Before evaluating the model on the independent external dataset, its performance was assessed using the internal testing subset derived from the first three datasets. None of the testing images had been previously presented to the network during training or validation, ensuring an unbiased internal evaluation of the proposed model.

**Figure 3:**
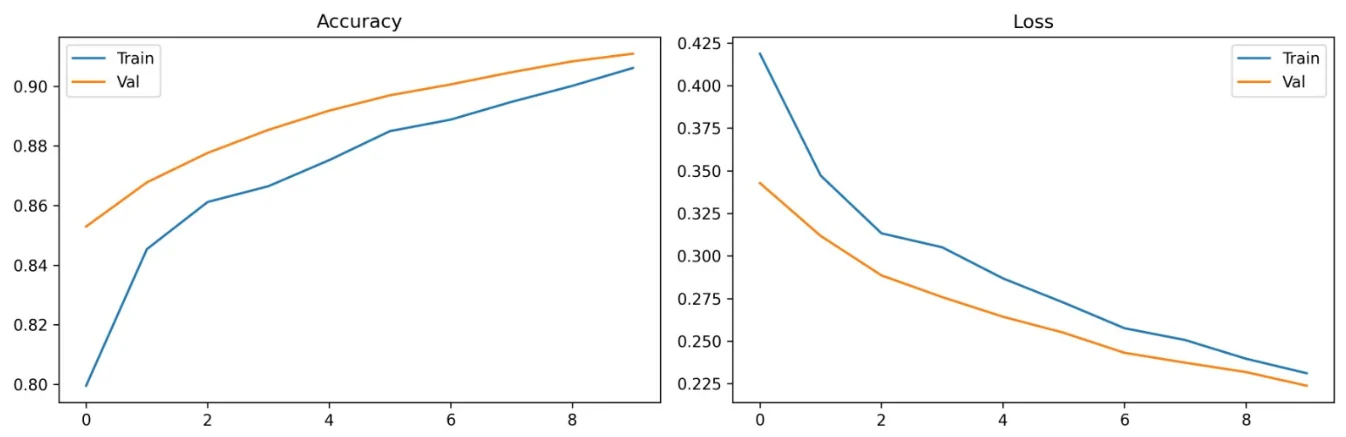
Training and validation accuracy and loss curves during model training.

During training, the model converged rapidly, reaching a final training accuracy of 89.8% and a validation accuracy of 91.0%. The corresponding binary cross-entropy loss decreased to 0.245 for the training set and 0.230 for the validation set, with no marked divergence between the training and validation curves (Figure 3).

Evaluation on the independent testing subset yielded an overall accuracy of 91.48%. According to the confusion matrix (Table 2), the proposed model correctly classified 2744 of 2952 malignant images and 1517 of 1706 normal images. The resulting sensitivity, specificity, precision, and F1-score were 92.95%, 88.92%, 93.56%, and 93.25%, respectively. The corresponding Youden’s J index was 0.819, reflecting the combined sensitivity and specificity of the model on the internal testing dataset. Matthews correlation coefficient (MCC) was calculated to complement the conventional performance metrics, yielding a value of 0.817 (CI95% = 0.800–0.834).

**Figure 4:**
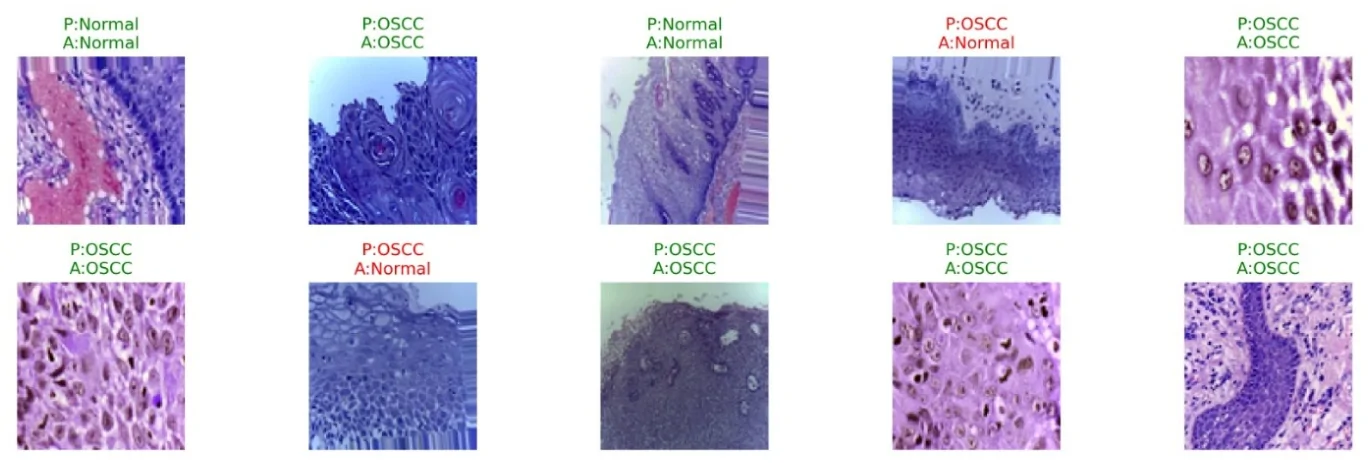
Representative predictions on the internal testing dataset, including correctly classified images and two false-positive examples.

Representative examples of correctly and incorrectly classified histopathological images are presented in Figure 4.

### 3.2. External Validation on an Independent Lymph Node Tissue Dataset

To assess the generalizability of the proposed model beyond the data used during model development, external validation was performed using an independent dataset composed of normal and metastatic lymph node tissue patches. Unlike the development datasets, which contained primary oral and oropharyngeal squamous cell carcinoma images, the external dataset consisted of downsampled (96 × 96 pixels) H&E-stained lymph node image patches acquired using a different image generation pipeline and tissue origin. None of these images were used during model training, validation, or internal testing.

**Figure 5:**
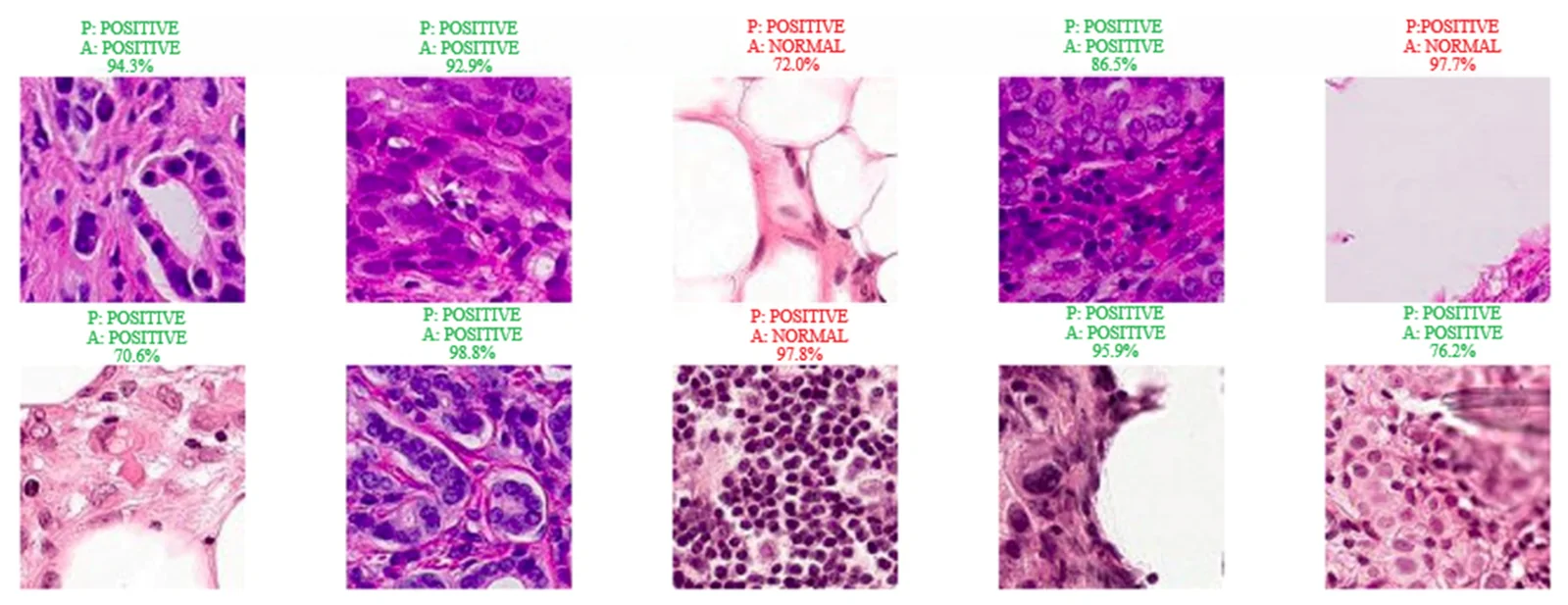
Representative positive predictions from the external validation dataset, including correctly classified and false-positive examples as well as a low-tissue-content patch illustrating a limitation of patch-level inference. The model-predicted probability for the positive class is displayed for each image.

The external validation was performed on a randomly selected subset of 20,000 labeled images. According to the confusion matrix (Table 3), the model correctly classified 7457 of 8225 metastatic tissue images, corresponding to a sensitivity of 90.66% (CI95% = 56.22–57.59%). Additionally, 3924 of 11,775 normal lymph node images were correctly identified, yielding a specificity of 33.32% (CI95% = 32.48–34.18%). The Youden’s J index obtained on the external validation dataset was 0.240. Figure 5 presents sample prediction examples from the external validation dataset.

Overall, the model achieved an accuracy of 56.91% (CI95% = 56.22–57.59%), precision of 48.71% (CI95% = 47.92–49.51%), F1-score of 63.34% (CI95% = 62.65–64.11%), and a balanced accuracy of 61.99% (CI95% = 61.46–62.52). The proposed model maintained a high sensitivity for metastatic tissue detection while exhibiting a substantial number of false-positive predictions among normal lymph node images.

MCC on the external validation dataset yielded a value of 0.279 (CI95% = 0.267–0.290).

These findings indicate that the model preserved a high ability to detect metastatic tissue despite the considerable differences between the training datasets and the external validation dataset in terms of tissue origin, image resolution, and acquisition methodology.

Uncertainty around classification metrics was estimated using non-parametric bootstrap resampling with 10,000 iterations. Bootstrap standard deviations and percentile-based 95% confidence intervals were calculated for accuracy, sensitivity, specificity, precision, F1-score, balanced accuracy, and MCC and are presented in Table 4 for both internal and external validation.

## 4. Discussions

The proposed model achieved an overall accuracy of 91.48%, with a sensitivity of 92.95%, specificity of 88.92%, precision of 93.56%, and an F1-score of 93.25% on the combined testing set of the first three datasets. Although higher classification accuracies have been reported in studies trained and evaluated on individual public datasets [23,24], achieving near-perfect performance was not the primary objective of the present work. Instead, the model was intentionally designed to learn robust morphological characteristics of oral squamous cell carcinoma across heterogeneous image sources rather than dataset-specific visual patterns. Consequently, no attempt was made to maximize performance through extensive architecture optimization or dataset-specific fine-tuning. The obtained performance was considered sufficient to provide a reliable feature representation while preserving the model’s ability to generalize to previously unseen data. This strategy was particularly important because the primary aim of the study was to evaluate the model on an independent fourth dataset originating from a different source, thereby assessing cross-dataset generalizability rather than maximizing performance on the training datasets alone.

Moreover, the first three datasets differed substantially in image acquisition characteristics, including image quality, color distribution, acquisition hardware, and the presence of acquisition-related artifacts. Training on such heterogeneous data inevitably represents a more challenging classification task than training on a single, homogeneous dataset. Nevertheless, exposing the model to this variability was expected to improve its robustness and reduce overfitting to dataset-specific characteristics, providing a more realistic evaluation of whether the learned representations remained transferable across heterogeneous image sources.

Although the proposed CNN demonstrated excellent performance during internal validation, a substantial decrease in performance was observed when evaluated on the independent lymph node dataset. This finding was expected, as the external validation represented a true out-of-distribution scenario. Unlike the development datasets, which consisted of primary OSSC and OPSSC, the external dataset contained lymph node tissue characterized by a completely different histological architecture, image resolution, acquisition pipeline, and tissue microenvironment. Consequently, the external validation assessed not only the ability of the model to recognize malignant morphology but also its capacity to generalize across different histopathological domains [25].

Despite these differences, the proposed model maintained a sensitivity exceeding 90%, indicating that many of the learned image features remained relevant for identifying metastatic tumor deposits. This observation suggests that the network did not rely exclusively on dataset-specific visual characteristics but instead learned morphological patterns commonly associated with malignant epithelial proliferation. Such features may include increased nuclear density, nuclear pleomorphism, irregular nuclear contours, hyperchromasia, architectural disorganization, and altered nucleus-to-cytoplasm ratio, all of which are frequently encountered in invasive squamous cell carcinoma and may also be present within metastatic epithelial deposits.

In contrast, specificity remained relatively low because a considerable proportion of normal lymph node patches were classified as metastatic tissue. Youden’s J decreased from 0.819 during internal testing to 0.240 during external validation, quantitatively illustrating the marked reduction in the model’s combined sensitivity-specificity performance under domain shift. One plausible explanation is the intrinsic histological composition of lymphoid tissue. Normal lymph nodes contain densely packed lymphocytes, resulting in high nuclear cellularity and intense hematoxylin staining throughout the tissue. These characteristics partially overlap with some of the low-level image features commonly associated with malignant tissue, particularly when image classification is performed on small patches without broader architectural context. Consequently, the network may have interpreted highly cellular but physiologically normal lymphoid tissue as malignant.

The high sensitivity observed during external validation should not be interpreted as evidence of immediate clinical applicability. Although a sensitivity of 90.66% indicates that most metastatic patches were identified, the corresponding specificity of 33.32% resulted in a substantial number of false-positive predictions. In a clinical screening or diagnostic-support setting, such a false-positive burden would markedly increase the number of cases requiring expert review and could reduce workflow efficiency. Therefore, the present model should be regarded as an experimental framework for investigating cross-domain transferability rather than a clinically deployable metastasis detector. Further optimization, including threshold calibration, domain-adaptation strategies, incorporation of normal lymph node morphology during training, and prospective validation on whole-slide images, would be required before considering clinical implementation.

The external validation represented a substantial out-of-distribution shift, with several variables changing simultaneously between the development and external datasets, including tissue type and histological architecture, image resolution and magnification, scanner characteristics, and patch-generation methodology. Consequently, the present experimental design does not allow the individual contribution of each factor to the observed reduction in specificity to be isolated. The decline in performance should therefore be interpreted as the combined effect of a complex domain shift rather than attributed to any single technical or morphological characteristic.

To complement conventional performance metrics, the MCC was also calculated, yielding a value of 0.279 on the external validation dataset. Since MCC simultaneously accounts for true-positive, true-negative, false-positive, and false-negative predictions, it provides a balanced assessment of binary classification performance even when the distribution of prediction errors is asymmetric. The positive but relatively modest MCC observed in the present study is consistent with partial discriminative ability under out-of-distribution conditions while also reflecting the substantial number of false-positive classifications. Together with the balanced accuracy of 61.99%, this finding further indicates that the preserved sensitivity should not be interpreted as uniformly strong external classification performance. The asymmetric external error pattern was further reflected by a false-positive rate of 66.68%, compared with a false-negative rate of only 9.34%, confirming that the decrease in external performance was predominantly driven by overclassification of normal lymph node tissue rather than failure to detect metastatic patches.

The positive class included well-, moderately-, and poorly differentiated squamous cell carcinomas. Among these, poorly differentiated tumors are generally characterized by reduced keratinization, increased cellularity, and marked nuclear atypia. Some of these characteristics overlap with morphological features encountered in metastatic epithelial tissue, including increased nuclear density, pleomorphism, hyperchromasia, altered nucleus-to-cytoplasm ratio, and architectural disorganization [25]. Therefore, the inclusion of tumors across different degrees of differentiation may have increased the morphological heterogeneity represented during training. However, the present external dataset does not provide tumor-grade information at the patch level, preventing a direct assessment of whether representations derived from poorly differentiated SCC contributed preferentially to metastatic tissue detection. This interpretation should therefore be regarded as hypothesis-generating rather than as a demonstrated mechanism underlying the observed sensitivity.

Interestingly, some false-positive examples received very high predicted probabilities, suggesting that the substantial domain shift may also affect model calibration. However, as prediction scores were not systematically analyzed across the entire external dataset, this observation remains exploratory and warrants dedicated calibration analysis in future studies.

The use of 96 × 96-pixel image patches may have contributed to the high false-positive rate observed in normal lymph node tissue. Image resizing inevitably reduces fine histomorphological detail, while patch-level classification limits access to the broader tissue architecture routinely used by pathologists for interpretation. This limitation may be particularly relevant in lymph nodes, where densely packed lymphocytes can produce high nuclear cellularity and intense hematoxylin-rich texture within small fields of view, potentially resembling some low-level features associated with malignant tissue. Nevertheless, image dimensions alone are unlikely to explain the observed decline in specificity, since the development images were also resized to 96 × 96 pixels while maintaining substantially higher internal specificity. The external performance degradation therefore most likely reflects the combined effects of reduced architectural context and the broader histopathological domain shift.

Besides the substantial differences in tissue architecture, the external validation dataset was also affected by potential scanner-related domain shift. Previous studies have demonstrated that variations in scanner manufacturers, image acquisition protocols, color reproduction, and batch effects significantly influence CNN performance and remain major obstacles to robust model generalization in computational pathology [26].

The present findings are consistent with previous work on domain generalization in computational pathology. The MIDOG 2022 challenge demonstrated that deep learning models retain the ability to transfer learned representations across different histopathological domains, although performance decreases when confronted with previously unseen tissue morphologies, scanners, or image acquisition protocols [27]. Likewise, recent studies have shown that inter-institutional variations in tissue processing, staining, slide digitization, and image acquisition remain major sources of domain shift, highlighting the need for dedicated domain generalization strategies [28]. These observations support our findings, where the transition from primary oral squamous cell carcinoma images to an independent lymph node tissue dataset resulted in reduced classification performance despite preserving high sensitivity for metastatic tissue detection.

Recent pathology foundation models have substantially advanced representation learning in computational pathology. Models such as H-Optimus-1 and GenBio-PathFM employ large Vision Transformer architectures and self-supervised pretraining to learn broadly transferable histopathological representations from highly diverse image collections. H-Optimus-1 was pretrained on more than one million whole-slide images spanning numerous organs, clinical centers, and scanner types using a DINO-based self-supervised strategy, whereas GenBio-PathFM combines DINO-based representation learning with a subsequent JEPA-based refinement stage and emphasizes automated selection of morphologically diverse training data [29,30]. However, H-Optimus-1 is currently described primarily through a conference abstract, whereas GenBio-PathFM remains available as a non-peer-reviewed preprint. Therefore, although these models represent important contemporary developments in computational pathology, direct benchmarking against large-scale pathology foundation models was not an objective of the present experimental study.

### 4.1. Limitations

Several limitations should be acknowledged. First, the study relied exclusively on publicly available datasets. Although these resources facilitate reproducibility, they frequently lack detailed metadata regarding patient characteristics, acquisition protocols, microscope or scanner models, staining procedures, and labeling methodology. In particular, the second dataset did not provide information regarding the imaging system, magnification verification, patient cohort, or annotation procedure, representing an important methodological limitation.

Second, all images were resized to 96 × 96 pixels to match the dimensions of the external validation dataset. Although this preprocessing step ensured methodological consistency, it inevitably reduced fine morphological detail that could potentially improve classification performance.

Furthermore, the proposed model was developed using isolated image patches rather than WSI. Consequently, the network relied exclusively on local cellular morphology and did not benefit from the broader architectural context routinely used by pathologists during histopathological diagnosis. Although three independent datasets were combined to improve data diversity and reduce overfitting to a single source, they all represented primary oral and oropharyngeal lesions. Consequently, tissue diversity remained limited, and the model was not exposed during training to the normal histological architecture of lymph nodes or other tissue types. Thus, patient-level independence could not be verified.

Since the ImageNet-pretrained backbone was used as a fixed feature extractor, the present study cannot determine whether similar representations could have been learned by training EfficientNetB0 de novo on the available histopathological data. The classification head was trained using a fixed 10-epoch schedule without validation-based early stopping or systematic checkpoint optimization; therefore, the potential effect of longer training or alternative checkpoint selection on internal and external performance was not assessed.

Another limitation concerns the external validation dataset. Although publicly available, detailed metadata regarding the primary tumor origin of metastatic lesions were unavailable. Consequently, subgroup analyses according to tumor type could not be performed, and the extent to which model performance differs across various metastatic carcinomas remains unknown.

Additionally, bootstrap-based uncertainty estimates were calculated at the patch level, as patient- or whole-slide-level identifiers were not available for the evaluated subset. Consequently, potential intra-slide correlations could not be accounted for, and the reported confidence intervals may underestimate uncertainty at the patient level.

The preserved external sensitivity suggests that some discriminative information learned from the primary SCC datasets remained transferable to the lymph node domain; however, the present study cannot determine whether these representations primarily reflect biologically relevant morphology or dataset- and acquisition-specific image characteristics.

Importantly, the present model should be regarded as an experimental framework for evaluating cross-domain transferability rather than as a clinically deployable diagnostic tool, as the high false-positive rate observed during external validation would currently preclude routine clinical use.

Finally, only a single CNN architecture (EfficientNetB0) was evaluated. Although EfficientNet has demonstrated excellent performance in numerous image classification tasks, future studies should compare multiple architectures and investigate domain adaptation or self-supervised learning strategies specifically designed to improve cross-domain generalization.

### 4.2. Future Work

Future work will focus on constructing a prospective institutional dataset of whole-slide images containing metastatic squamous cell carcinoma within cervical lymph nodes. Such a dataset would allow evaluation under standardized acquisition conditions, preserve full image resolution, provide complete clinicopathological metadata, and facilitate further investigation of cross-domain feature transfer using both patch-based and whole-slide approaches. Moreover, this type of approach could be expanded to other types of carcinomas such as nasopharyngeal carcinoma [31].

Future studies should also extend the present experimental framework using prospectively curated and fully traceable histopathological datasets with patient- and WSI-level identifiers. Such data would enable rigorous linear-probe analyses without source-slide leakage, stratified evaluation according to primary tumor origin and histological differentiation, and direct assessment of whether specific SCC phenotypes contribute preferentially to cross-domain transferability. Additional experiments should also investigate the contribution of ImageNet initialization through controlled comparisons with randomly initialized and fully trained networks, as well as longer training schedules, validation-based checkpoint selection, and backbone fine-tuning. Dedicated domain-generalization approaches, including stain normalization, domain adaptation, and tissue-content filtering, should be evaluated together with formal probability-calibration and decision-threshold analyses. Moreover, comparison with contemporary pathology-specific foundation models may help determine whether large-scale self-supervised histopathological pretraining provides greater robustness under severe domain shift than conventional ImageNet-based feature extraction. Finally, future work should incorporate whole-slide image analysis and interpretability or feature-attribution methods to better characterize the morphological information driving model predictions and to distinguish biologically relevant tissue representations from scanner-, staining-, or dataset-specific characteristics. These directions may provide a more rigorous understanding of both the potential and the limitations of representation transfer across distinct histopathological domains.

## 5. Conclusions

The proposed EfficientNetB0-based classifier demonstrated strong performance within the development domain, achieving an internal testing accuracy of 91.48%, sensitivity of 92.95%, specificity of 88.92%, and F1-score of 93.25%. These results indicate that the ImageNet-pretrained EfficientNetB0 backbone, used as a fixed feature extractor together with a task-specific classification head, provided effective discrimination between normal and malignant oral/oropharyngeal histopathological image patches within the evaluated datasets.

External validation on an independent lymph node histopathology dataset resulted in a substantial decrease in overall performance, with an accuracy of 56.91%, specificity of 33.32%, balanced accuracy of 61.99%, and MCC of 0.279. Nevertheless, sensitivity for metastatic tissue remained high at 90.66%. The asymmetric external performance, characterized by preservation of sensitivity but a high false-positive rate, indicates that some discriminative information remained transferable across domains while also demonstrating that the learned representations were insufficiently specific for robust classification within the markedly different lymph node tissue environment. The present findings therefore support partial cross-domain transferability rather than universal generalization and should not be interpreted as evidence of clinical readiness.

Overall, this study highlights both the potential and the limitations of deep learning representation transfer under substantial histopathological domain shift. Evaluation on independent out-of-distribution datasets provides information that cannot be captured by internal testing alone and may reveal important limitations in model robustness. Future studies using prospectively curated whole-slide image datasets of metastatic cervical lymph nodes, complete patient- and slide-level metadata, standardized acquisition protocols, pathology-specific pretraining, domain-adaptation strategies, and dedicated representation analyses will be required to further characterize and improve cross-domain generalization in computational pathology.

## Figures and Tables

**Table 1:** Distribution of histopathological images across the three datasets used for model development.

	1st Database	2nd Database	3rd Database
**SCC**	2634	2501	9129
**Normal**	2494	2501	1035

**Table 2:** Confusion matrix obtained on the internal testing dataset derived from the first three histopathological databases.

	Predicted Normal	Predicted OSCC	Total
**Actual Normal**	1517	189	1706
**Actual OSCC**	208	2744	2952
**Total**	1725	2933	4658

**Table 3:** Confusion matrix obtained on the independent external lymph node tissue dataset.

	Predicted Normal	Predicted Metastasis	Total
**Actual Normal**	3924	7851	11,775
**Actual Metastasis**	768	7457	8225
**Total**	4692	15,308	20,000

**Table 4:** Performance metrics obtained on the internal testing and external validation datasets.

Metric	Estimate	Bootstrap SD	95% CI
**Internal testing**			
**Accuracy**	91.48%	0.41 pp	90.68–92.25%
**Sensitivity**	92.95%	0.47 pp	92.04–93.87%
**Specificity**	88.92%	0.76 pp	87.43–90.38%
**Precision**	92.56%	0.45 pp	92.65–94.43%
**F1-score**	93.25%	0.33 pp	92.61–93.89%
**Balanced accuracy**	90.94%	0.44 pp	90.06–91.79%
**MCC**	0.817	0.009	0.800–0.834
**External Validation**			
**Accuracy**	56.91%	0.35 pp	56.22–57.61%
**Sensitivity**	90.66%	0.32 pp	90.03–91.28%
**Specificity**	33.32%	0.43 pp	32.49–34.19%
**Precision**	48.71%	0.41 pp	47.92–49.52%
**F1-score**	63.37%	0.37 pp	62.65–64.11%
**Balanced accuracy**	61.99%	0.27 pp	61.46–62.52%
**MCC**	0.279	0.006	0.267–0.290

## Data Availability

All data are available on reasonable request from the corresponding authors.

## Supplementary Materials

The following supporting information can be downloaded at: https://www.mdpi.com/article/10.3390/cancers18172752/s1, File S1: The AI code used.

## Abbreviations

The following abbreviations are used in this manuscript:

AI	Artificial intelligence
CNN	Convolutional neural network
H&E	Hematoxylin and eosin
HNSCC	Head and neck squamous cell carcinoma
HPV	Human papillomavirus
IoU	Intersection over Union
MDSCC	Moderately differentiated squamous cell carcinoma
OOD	Out-of-distribution
OPSCC	Oropharyngeal squamous cell carcinoma
OSCC	Oral squamous cell carcinoma
OSMF	Oral submucous fibrosis
PDSCC	Poorly differentiated squamous cell carcinoma
Rb	Retinoblastoma
ReLU	Rectified Linear Unit
SCC	Squamous cell carcinoma
TAN	Tumor-associated neutrophil
WDSCC	Well-differentiated squamous cell carcinoma
WSI	Whole-slide image
WSIs	Whole-slide images
