# Supplementary material for: Cross-Domain Generalization of a CNN Trained on Oral and Oropharyngeal Squamous Cell Carcinoma Histopathology Using External Validation on Metastatic Lymph Node Tissue
Source: Cancers (Basel). 2026 Aug 25;18(17):2752. doi: 10.3390/cancers18172752 (PMC13565022; doi:10.3390/cancers18172752)
Supplement: Supplementary file 1 [file cancers-18-02752-s001.zip › cancers-4512235-supplementary-File S1.pdf]

1. TRAINING AND INTERNAL TESTING

```
import numpy as np
import os

os.environ['TF_CPP_MIN_LOG_LEVEL'] = '3'
os.environ["TF_XLA_FLAGS"] = "--tf_xla_enable_xla_devices=false"

import random
import tensorflow as tf
from sklearn.model_selection import train_test_split
from tensorflow.keras.applications import EfficientNetB0
from tensorflow.keras import layers, models
from tensorflow.keras.applications.efficientnet import preprocess_input
from sklearn.metrics import classification_report, confusion_matrix
import matplotlib.pyplot as plt
import seaborn as sns
from collections import Counter
from PIL import Image
import gc
from tqdm.auto import tqdm
import warnings

random.seed(42)
np.random.seed(42)
tf.random.set_seed(42)
warnings.filterwarnings('ignore')

Histopatological_Cancer_Detection = " " #input database
Histopatological_Imaging_Dataset_for_Oral_Cancer = " " #input database
Oral_New = " " #input database
# oral_scc - 1 oral_normal - 0

train_images = []
dataset_1 = []
for split in ['train', 'val', 'test']:
    for label in ['OSCC', 'Normal']:
        folder = f"{Histopatological_Imaging_Dataset_for_Oral_Cancer}/{split}/{label}"
        for img in os.listdir(folder):
            dataset_1.append((os.path.join(folder, img), label))
dataset_2 = []
label_map = {
    "oral_scc": "OSCC",
    "oral_normal": "Normal"
}
for split in ["train", "val", "test"]:
    for folder_name, label in label_map.items():
        folder = os.path.join(Cancer_Classification, split, folder_name)
if not os.path.isdir(folder):
            print(f"Folder not found: {folder}")
            continue
        for img in os.listdir(folder):
            img_path = os.path.join(folder, img)
            if os.path.isfile(img_path):
                dataset_2.append((img_path, label))

dataset_3 = []
label_map = {
    "normal": "Normal",
    "mdoscc": "OSCC",
```

```
    "pdoscc": "OSCC",
    "wdoscc": "OSCC"
}

for split in ["Train", "Test"]:
    for folder_name, label in label_map.items():

        folder = os.path.join(Oral_New, split, folder_name)

        if not os.path.isdir(folder):
            print(f"Folder inexistent: {folder}")
            continue

        for img in os.listdir(folder):
            img_path = os.path.join(folder, img)

            if os.path.isfile(img_path) and img.lower().endswith(
                (".jpg", ".jpeg", ".png", ".bmp", ".tif", ".tiff")
            ):
                dataset_3.append((img_path, label))

#Class distribution
normal = sum(label == "Normal" for _, label in dataset_3)
oscc = sum(label == "OSCC" for _, label in dataset_3)

train_images = dataset_1 + dataset_2 + dataset_3

#Memory release
del dataset_1, dataset_2, dataset_3
gc.collect()

processed_images = []
for img_path, label in tqdm(train_images, desc="Procesare imagini"):
    try:
img = Image.open(img_path).convert("RGB")
img = img.resize((96, 96), Image.Resampling.LANCZOS)
img = np.array(img, dtype=np.float32)
img = preprocess_input(img)
processed_images.append((img, label))

except Exception as e:
        print(f"\nError {img_path}: {e}")

train_images = processed_images
del processed_images
gc.collect()

#Divide images (X) from labels (Y)
X = np.array([img for img, label in train_images], dtype=np.float32)
y = np.array([
    1 if label == "OSCC" else 0
    for img, label in train_images
], dtype=np.int32)

print(f"X shape: {X.shape}")
print(f"y shape: {y.shape}")

#Split train, validation, test
X_train, X_temp, y_train, y_temp = train_test_split(
    X,
```

```
    y,
    test_size=0.40,
    random_state=42,
    stratify=y
)

X_val, X_test, y_val, y_test = train_test_split(
    X_temp,
    y_temp,
    test_size=0.50,
    random_state=42,
    stratify=y_temp
)

#Model training
base = EfficientNetB0(include_top=False, weights='imagenet', input_shape=(96,96,3))
base.trainable = False
model = models.Sequential([
    base,
    layers.GlobalAveragePooling2D(),
    layers.Dropout(0.3),
    layers.Dense(128, activation='relu'),
    layers.Dropout(0.2),
    layers.Dense(1, activation='sigmoid')
])
model.compile(
    optimizer='adamax',
    loss='binary_crossentropy',
    metrics=[
        'accuracy',
        tf.keras.metrics.Precision(name="precision"),
        tf.keras.metrics.Recall(name="recall"),
        tf.keras.metrics.AUC(name="auc")
    ]
)
history = model.fit(X_train, y_train, validation_data=(X_val, y_val), epochs=10)

#Test predictions
y_pred = (model.predict(X_test) > 0.5).astype(int).flatten()
y_true = y_test
print(classification_report(y_test, y_pred, target_names=['Normal', 'OSCC']))

#Results
plt.figure(figsize=(12,4))

plt.subplot(1,2,1)
plt.plot(history.history['accuracy'], label='Train')
plt.plot(history.history['val_accuracy'], label='Val')
plt.title('Accuracy')
plt.legend()

plt.subplot(1,2,2)
plt.plot(history.history['loss'], label='Train')
plt.plot(history.history['val_loss'], label='Val')
plt.title('Loss')
plt.legend()

plt.tight_layout()
plt.savefig("training_curves.png", dpi=300)
plt.show()
```

```
cm = confusion_matrix(y_true, y_pred)

plt.figure(figsize=(6,5))
sns.heatmap(
    cm,
    annot=True,
    fmt='d',
    cmap='Blues',
    xticklabels=['Normal','OSCC'],
    yticklabels=['Normal','OSCC']
)

plt.xlabel('Predicted')
plt.ylabel('Actual')
plt.title('Confusion Matrix - Test Set')

plt.savefig("confusion_matrix.png", dpi=300, bbox_inches='tight')
plt.show()

class_names = ['Normal', 'OSCC']

save_path = "sample_predictions_grid.png"

idx = np.random.choice(len(X_test), 10, replace=False)

images = X_test[idx]
labels = y_test[idx]

preds = (model.predict(images) > 0.5).astype(int).flatten()

plt.figure(figsize=(15,5))

for i in range(10):
    plt.subplot(2,5,i+1)

    plt.imshow(images[i].astype("uint8"))

    color = 'green' if preds[i] == labels[i] else 'red'

    plt.title(
        f'P:{class_names[preds[i]]}\nA:{class_names[labels[i]]}',
        color=color
    )
    plt.axis('off')

plt.suptitle('Sample Predictions (Green=Correct, Red=Wrong)')

plt.tight_layout()
plt.savefig(save_path, dpi=300, bbox_inches='tight')
plt.close()

#Saving model
model.save("model.keras")
```

2. EXTERNAL VALIDATION

```
import matplotlib.pyplot as plt
import numpy as np
import os
import pandas as pd
from tensorflow.keras.models import load_model
from PIL import Image
from tqdm.auto import tqdm
import random

DATASET_PATH = " " #input database 4

model = load_model () #Input previous trained model
model.summary()

dataset_test = []
labels_df = pd.read_csv(
    os.path.join(DATASET_PATH, "train_labels.csv")
train_folder = os.path.join(DATASET_PATH, "train")
for _, row in labels_df.iterrows():
        img_path = os.path.join(train_folder, f"{row['id']}.tif")
        label = "positive" if row["label"] == 1 else "Normal"
        if os.path.isfile(img_path):
          dataset_test.append((img_path, label))
print(f"Total number of images: {len(dataset_test)}")

#Class distribution
normal = sum(label == "Normal" for _, label in dataset_test)
positive = sum(label == "positive" for _, label in dataset_test)
for sample in dataset_test[:5]:
    print(sample)

#Random images selector
random.seed(42)
dataset_test_sample = random.sample(dataset_test, 20000)
X_dataset_test = []
y_dataset_test = []
for img_path, label in tqdm(dataset_test_sample, desc="proc dataset test"):
    try:
        img = Image.open(img_path).convert("RGB")
        img = img.resize((96, 96), Image.Resampling.LANCZOS)
        img = np.array(img, dtype=np.float32)
        X_dataset_test.append(img)
        y_dataset_test.append(1 if label == "positive" else 0)
    except Exception as e:
        print(f"Eroare la {img_path}: {e}")

X_dataset_test = np.array(X_dataset_test, dtype=np.float32)
y_dataset_test = np.array(y_dataset_test, dtype=np.int32)

#Metrics
from sklearn.metrics import (
    confusion_matrix,
    classification_report,
    accuracy_score,
    precision_score,
    recall_score,
```

```
    f1_score,
)
y_pred_prob = model.predict(X_dataset_test, batch_size=32, verbose=1)
y_pred = (y_pred_prob >= 0.5).astype(int).flatten()
accuracy = accuracy_score(y_dataset_test, y_pred)
precision = precision_score(y_dataset_test, y_pred)
recall = recall_score(y_dataset_test, y_pred)
f1 = f1_score(y_dataset_test, y_pred)
cm = confusion_matrix(y_dataset_test, y_pred)
tn, fp, fn, tp = cm.ravel()
specificity = tn / (tn + fp)

print("=" * 50)
print("REZULTATE PE DATASET 4")
print("=" * 50)
print(f"Accuracy   : {accuracy:.4f}")
print(f"Precision  : {precision:.4f}")
print(f"Recall     : {recall:.4f}")
print(f"Specificity: {specificity:.4f}")
print(f"F1-score   : {f1:.4f}")
print(f"ROC AUC    : {auc:.4f}")

#Examples
class_names = ["Normal", "Positive"]
idx = np.random.choice(len(X_dataset_test), 10, replace=False)
images = X_dataset_test[idx]
labels = y_dataset_test[idx]
pred_probs = model.predict(images, verbose=0)
preds = (pred_probs >= 0.5).astype(int).flatten()
plt.figure(figsize=(15, 6))
for i in range(10):
   plt.subplot(2, 5, i + 1)
   img = images[i]
   if img.max() <= 1.0:
           img = (img * 255).astype(np.uint8)
   else:
           img = np.clip(img, 0, 255).astype(np.uint8)
   plt.imshow(img)

   color = "green" if preds[i] == labels[i] else "red"
       confidence = pred_probs[i][0] * 100
       if preds[i] == 0:
           confidence = 100 - confidence
       plt.title(
           f"P: {class_names[preds[i]]}\n"
           f"A: {class_names[labels[i]]}\n"
           f"{confidence:.1f}%",
           color=color,
           fontsize=10
       )
       plt.axis("off")
   plt.tight_layout()
   plt.savefig("dataset4_sample_predictions.png", dpi=300, bbox_inches="tight")
   plt.show()
```
